# Reflections on the “gesture-first” hypothesis of language origins

**DOI:** 10.3758/s13423-016-1117-3

**Published:** 2016-07-20

**Authors:** Adam Kendon

**Affiliations:** 1 Psychology and Language Sciences, University College London, London, UK; 2 Division of Biological Anthropology, Cambridge University, Cambridge, UK; 3 2, Orchard Estate, Cherry Hinton, Cambridge CB1 3JP UK

**Keywords:** Gesture, Language origins, Speech, Primate communication, Sign language

## Abstract

The main lines of evidence taken as support for the “gesture-first” hypothesis of language origins are briefly evaluated, and the problem that speech poses for this hypothesis is discussed. I conclude that language must have evolved in the oral–aural and kinesic modalities together, with neither modality taking precedence over the other.

## “Gesture first”: Origins and difficulties

The idea that humans were first able to communicate in a symbolic way by gesture, and so were able to develop language, has a long history (Hewes, 1999). From the beginning of the eighteenth century onward, when the natural origins of language began to be discussed, the “gesture-first” idea was put forward by several prominent thinkers—for example, by Étienne Bonnot de Condillac, in Paris in 1746 (Condillac, 2001), or by Giambattista Vico, in Naples in 1744 (Bergin & Fisch, 1984). It was further sympathetically discussed in the nineteenth century by Edward Tylor (1865), Garrick Mallery (1881/1972), George Romanes (1898), and Wilhelm Wundt (1901/1973), among others.

By the end of the nineteenth century, discussion of language origins went into eclipse (Stam, 1976), but it began to revive again in the late 1960s. An important contribution to this revival was Hewes (1973), an article published in *Current Anthropology* that attracted considerable commentary. Within 2 years, a large conference on the question was held at the New York Academy of Sciences (Harnad, Steklis, & Lancaster, 1976), since which time the issue has become a major focus of academic activity.

Hewes’s 1973 article, which made a strong case for the gesture-first hypothesis, was in part inspired by Beatrice and Allen Gardner’s success in teaching a chimpanzee to use manual actions based on signs from American Sign Language in apparently symbolic ways (Gardner & Gardner, 1969). As compared to previous attempts to teach apes to speak, which had been failures (Hayes, 1951; Kellogg & Kellogg, 1933), the success of the Gardners’ project seemed spectacular. It seemed to be a challenge to the idea that the Rubicon that separates man from other animals and that, as Max Müller declared, “no brute will dare to cross” (Müller, 1868, p. 354). For this reason, it now seemed very important to study the nature of sign language, to determine its linguistic status. Research proposed by Jacob Bronowski (Bronowski & Bellugi, 1970), begun under Ursula Bellugi at the Salk Institute in San Diego, at first aimed to investigate children learning sign language to compare with Washoe. Soon it was realized that the fundamental nature of sign language needed investigation. *The Signs of Language* (Klima & Bellugi, 1979), presenting results from the first decade of this work, confirmed and amplified that sign language is in every way a proper language. Stokoe (1960) had already shown this, but he received little attention until the new initiative in the study of sign language began a decade later.

Since Hewes’s article, the gesture-first hypothesis has been discussed and supported by many others (e.g., Arbib, 2012; Armstrong, Stokoe, & Wilcox 1995; Corballis, 2002; Stokoe, 2002; Tomasello, 2008). In what follows, I briefly review the evidence commonly appealed to as supporting “gesture first,” but argue that it is far from conclusive. I discuss the problem that human specialization as a speaking animal raises for any gesture-first hypothesis, but also point out the importance of taking into account the phenomena of co-speech gesturing in any theory of language origins.

## Evidence for “gesture first”: Summary and critique

1. *Vocalization in nonhuman primates.* Since, for a long time, it was understood that ape and monkey vocal communication involved only a fixed repertoire of inarticulate calls, under limited voluntary control, it was thought unlikely that language would have evolved from this.
  However, recent work on nonhuman primate vocalization has shown that in many species it is much more flexible, complex, and articulated than was previously supposed (see Lemasson, 2011; Zuberbühler, Arnold, & Slocombe, 2011). Vocalizing in combination with tongue and lip displays such as lip-smacking, used in close and friendly interactions, can suggest a model of how speech could have derived from oral–aural actions. Although only humans have voluntary control of the larynx, mediated by a direct connection to the cortex (Jurgens, 2000; see also the review in Fitch, 2010, chap. 9), in the nonhuman primate species that have been investigated (notably the squirrel monkey; see Jurgens, 1998), the lips and tongue are controlled by such a direct connection, and their movements are under voluntary control. Combining visible displays of lips and tongue with vocalizing already can make possible the production of a complex range of sounds. It is not difficult to imagine the further modifications of neural control that would be needed for the full voluntary control of articulated sound that the development of speech would require (see Fitch, 2010, for a review). It is notable that studies by Bergman (2013) and by Ghazanfar, Takahashi, Mathur, and Fitch (2012) have shown that lip-smacking in geladas is similar to human speech in its rhythmicity and developmental trajectory, and in how the lips and tongue are coordinated. All of this contributes to the idea that the articulatory complexities of speech may have eventually developed from mouth and tongue displays of this sort.
2. *Ape gestures.* In contrast, the flexibility and learnability of forelimb gestures, especially in the great apes, makes this modality seem more amenable as a medium in which language could have first emerged (Pollick & de Waal, 2007). So far, however, although ape gesturing may be comparable to human gestures serving in the management of interpersonal relations—greeting, beckoning, offering, rejecting, and so forth—gestures of a depictive or referential nature have not been reliably observed. As far as is known, in captivity apes may engage in imperative pointing, but not in declarative pointing, which human children do from an early age.
3. *Ape language experiments.* The discovery that apes could be taught to use hand gestures derived from sign language in apparently symbolic ways seemed to confirm the idea that gesture could have been the first language medium, as was already mentioned (Wallman, 1992, pp. 10–28, describes ape language projects up to 1990). However, apes only learn to use signs symbolically under the very special conditions of close and continuous relationship with humans. This work suggests a symbolic capacity in apes, but tells little of how this capacity came to be expressed naturally—if, in fact, it ever did.
4. *Ontogenesis of speech and gesture.* Studies of very young children have suggested that pointing, followed by symbolic gestures, preceded the acquisition of speech. This has been considered compatible with a gesture-first position (e.g., Meguerditchian, Cochet, & Vauclair, 2011)
  Before the observations on the apparent prespeech appearance of pointing and symbolic gestures can be accepted as support for gesture preceding speech phylogenetically, however, more will need to be understood about whether and how vocalizations accompany these actions. “Babbling,” though it is not intelligible, constitutes an attempt at speech. As Trevarthen (1979) and others have observed, there is often close coordination between babbling and hand movements, as if efforts toward symbolic expression are being made both kinesically *and* orally. Cochet and Vauclair (2010), in an observational study of spontaneous declarative pointing in children between the ages of approximately 11 months and a little over 3 years, showed that most of the time these pointings were accompanied by vocalizations. A study by Grünloh and Liszkowski (2015) of the vocalizations that co-occur with pointing in prelinguistic infants showed that these may be differentiated in such a way that they indicate whether the pointing is imperative or declarative. Vocalizations before language, thus, may well be attempts at meaningful expression, even though the infant does not yet have command of any socially shared vocabulary. Such vocalizations deserve much more study, from this point of view, than they have hitherto received. Perhaps the appearance of intelligible gesture prior to intelligible speech has been reported because adults can more readily interpret semantically a baby’s kinesic expressions than than they can its oral expressions.
5. *Sign languages.* The fact that fully functional languages can develop in the kinesic medium—as in sign languages among the deaf—has been taken to support “gesture first.” Thus, Tomasello (2008, p. 328) stated that the readiness with which humans can create sign languages, as exemplified by the emergence of Nicaraguan Sign Language (Kegl, Senghas, & Coppola, 1999; Senghas, Senghas, & Pyers, 2005), would be “incredible, almost inexplicable . . . [if] humans were adapted for a vocal language only”; however, “[i]f humans were adapted first for something like gestural communication, and the vocal modality took over only later, then these gestural inventions are much more readily explained.” Perhaps so; however, it would have been just as persuasive if Tomasello had written, “if humans were *also* adapted for something like gestural communication.” As I will suggest again below, there seems to be no reason to suppose that the development of a capacity for symbolic expression was at first confined to only one modality.
  The study of sign languages is relevant to the problem of language origins, not because they might be models for early forms of language or may support Tomasello’s claim, but because we can sometimes observe their formation and the conditions under which this happens. For example, examining how signers invent new signs when they lack an expression for something allows us to witness the process through which linguistic symbols are created, and how they become transformed as they become socially shared (Tylor, 1865, made this point). As they become freed from depending upon iconicity for intelligibility, they can function as Saussurean elements within a diachronic system. When this is studied within the social conditions in which these transformations occur, insight is gained into the processes through which language as a socially shared system comes into being. Modern signers, however, as fully evolved humans for whom deafness is only an accidental feature, cannot throw light on earlier stages of language evolution.
6. *Speaker gesturing.* The complex use of manual gestures by speakers that are integrated with speech was said by Hewes (1973) to indicate that speech was a later addition to an already existing gesture system.
  However, the fact that speaker gesturing is fully integrated into the utterances of which it forms a part (Kendon, 2004, 2014; McNeill, 1992) suggests that spoken expression and manual expression evolved conjointly, not that gesturing persisted as a leftover that speech overlaid. As will be noted later, many speaker gestures appear to be derivatives of object-handling actions. This may suggest the original praxic nature of language, but not the primitiveness of gesture (see Kendon, 2009).
7. *Neurology.* Neurological investigations have shown that hand and mouth actions are controlled by very closely related systems. The detailed knowledge being developed about this underlines the co-involvement of hand and mouth, in both languaging and practical activities. Comparative studies should throw light on the evolutionary history of this partnership. At present, however, all we can say is that this supports arguments for the joint evolution of these systems as well as it supports arguments claiming the precedence of hand over mouth (see, e.g., Gentilucci & Dalla Volta, 2007; Kimura, 1993; Willems & Hagoort, 2007).
8. *Mirror neurons.* The discovery of mirror neurons was hailed by Rizzolatti and Arbib (1998) as offering a solution to what they called the “parity problem”—how conspecifics can mutually understand each others’ actions, whether communicative or praxic—a problem that would have to be solved to allow the development of language. Arbib has since developed his “mirror system hypothesis,” in which he supposes that language eventually became possible because of the mutual understanding of grasping actions made possible by the mirror system (Arbib, 2012). However, many problems remain regarding the nature and role of mirror neurons, whether in macaque monkeys or in humans. In particular, as Hickock (2009) pointed out, it is far from clear whether mirror neurons play any role in the processes through which individuals understand the actions of others. It seems much more likely that these processes would have more to do with the processes involved in an individual’s own motor control (Hickock, 2014). The claim that the discovery of mirror neurons offered strong support for the gesture-first view of language origins (e.g., Corballis, 2010) now seems overstated.

Although these eight lines of evidence may make a plausible case for the “gesture-first” view, in my opinion they do not make a compelling one. Furthermore, because humans are specialized as speaking animals (Ghazanfar & Rendall, 2008; Lenneberg, 1967), any “gesture-first” theory would remain incomplete until it could offer a satisfactory account of why this should be. The specializations for speaking, in regard to both the production of speech and its reception, are complex and extensive, and would have required a long period of time to evolve. This implies that the oral–aural modality must have long been involved in whatever changes in effecting communicative actions were taking place that ultimately gave rise to linguistic communication. We must suppose that these developments came about as part of a trend toward greater complexity in communication generally, changes that were probably linked to changes in the complexities of social organization (Freeberg, Dunbar, & Ord, 2012) and in the management of face-to-face interaction (Levinson, 2006). These changes, involving the oral–aural modality, would surely also have involved the kinesic modality. We may suppose, for example, that concomitant with developments in oral articulatory skill and the voluntary control of the larynx, changes would have taken place in the complexity and subtlety of control of the musculature of the human face, or changes such as those leading to the white sclera of the eyes becoming visible, a human-specific feature that facilitates the subtle detection of where another individual is looking (Kobayashi & Koshima, 2001). These are but two of the more obvious human features that appear to be specific adaptations for close and complex face-to-face interaction, involving many different action systems. The orchestration of these different systems using different modalities, which is so impressive a feature of human face-to-face interaction (see, e.g., Goodwin 2000), gives a strong reason to suppose that both vocal and kinesic forms of communication were intimately involved all along, as humans developed languaging.

In a recent article, written in part as a response to an earlier discussion of mine (Kendon, 2011), Michael Corballis (2014) offered a review of the evidence adduced in support of the “gesture-first” position in much the way I have tried to do here. He agreed with me that the evidence makes a plausible case for the “gesture-first” view, but for him it also supports this view, although he conceded that it does so only “fairly marginally” (p. 190). He remains persuaded by the “gesture-first” idea mainly because, despite recent evidence that apes do show some degree of voluntary control of their vocal productions, this remains very limited, and is in marked contrast to the versatility with which apes use gestures, often including ways that can almost be regarded as symbolic. In his view this makes it much more likely that language began with gestures, only later involving the oral–aural modality as well.

As Corballis pointed out, the difference between my view and his is not very great. Like him, as will be seen in the next section, I think the articulated vocalization that speech uses could have arisen through a development that involved the combining of lip and tongue displays with vocalization. I likewise agree with him that language is deeply connected to practical action and may be a derivation from it (a point to be discussed below). I also think that Corballis would agree that the key shift that was required to make practical actions (whether by mouth or by hand) available as material for something that could become language was that these actions should become able to refer to things not immediately present and be recognized as having this function. This would enable users to share imagined rather than real actions. Once this can happen, the way is open for dialogues about things imagined (cf. Kendon, 1991). This would be crucial if a system of communication were to develop into something we would regard as “language.” That Corballis (2013) would be sympathetic to this view is shown by his discussion of the importance of what he calls “mental time travel,” which, with language, makes it possible for us to share memories, plans, and ideas. However, unlike Corballis (2013), I think that the shift into symbolic use would have affected *both visible action and oral–aural action at the same time*. As sign languages and “picture writing systems” (semaisographic systems; Sampson, 1985) have taught us, you can build a language in any modality. All kinds of behavior can become symbolic. Given the intimate coordinated relationship between hand actions and the mouth actions of speaking, which we have already noted and must have been established at a very early stage in primate evolution (see Wise, 2009), it is not clear to me why, if a symbolic use of behavior became established, this would occur first only in visible bodily action, and not in oral–aural action as well.

## The question of speech

As we have already noted, humans, in various ways—neurological and anatomical—are specialized for the production and reception of speech. Gesture-first theorists have recognized this, but none have offered a good framework that can account for it. Some have asked: Why is it, if the ancestors of *Homo* developed a sign language, did they then change to a spoken language? There must have been a switch from one modality to the other (see Corballis, 2002; Hewes, 1973; Tomasello, 2008). What brought this about?

The answer has usually been to list the advantages of speech over gesture. For example, it is said to be faster than signing, and if speech is used, the hands can do other things at the same time, something that is not possible if the hands are busy signing. Speech can be understood in the dark, and it also allows for communication in environments where the parties cannot see one another—for example, in wooded areas.

Such advantages cannot serve to answer the question of why speech was selected. Evolution does not work in terms of possible but as-yet-unrealized advantages. It works only by gradual modifications of existing systems selected for their greater effectiveness for *current* functions. This can and does result in changes, allowing the modified system to be capable of new functions, which may then be selected for. The evolutionary changes that eventually led to speech did lead to new things along the way, but these new things did not guide the process.

The very question of a “switch” seems inappropriate, however, since it seems to imply that speech *or* gesture were already available possibilities and that a choice could have be made between them. This surely was never the case. As Darwin (1871, pp. 58–59) pointed out, “[a]s all the higher mammals possess vocal organs constructed on the same general plan as ours, and which are used as a means of communication, it was obviously probable, if the power of communication had to be improved, it would have been still further adapted.” Evolution builds on what is already there, and the vocal apparatus, already part of the mammalian plan and used as a system for communication, would have been the framework within which further developments in communicative mouth and voice actions would have taken place. Human speech must be the outcome of a very long process of modification to already-existing modes of vocal expression, developing from prior stages in which, as social life became more complex, increasingly complex articulated phonations were used, perhaps in chorusing, and in dialogic exchanges, whether playful, flirtatious, affiliative, competitive, or agonistic. If the oral–aural modality always was a component of such interactions along with kinesis, the issue of whether gesture or speech came first does not arise: Both modalities served communicatively from the beginning.

What drove the oral–aural modality to develop articulatory complexity? Comparative studies of several different species of monkeys have shown correlations between the complexity of vocal repertoires and the complexity of social organization (Gustison et al., 2012; McComb & Semple 2005). This has also been found in birds, squirrels, and bats (see the references in Freeberg, 2006). Similar correlations between the complexity of social life and the complexity and variety of facial displays in various species of monkey have been found by Maestripieri (1999, 2005) and by Dobson (2009). Freeberg, Dunbar, and Ord (2012) provide a comprehensive review and discussion of this point, proposing what they term the *social complexity hypothesis* for communication. This proposes that more complex social systems will require members to employ more complex communication systems. That is, there will be a wider and more diverse repertoire of communicative signals, both vocal and visible, in societies that are larger, have differentiated social roles and more complex interaction networks, and include maintained pair relationships (as between mates, but also long-term friend relationships), than in societies that have fewer of these features. This means, of course, that any evolutionary account of human language will have to take account of the evolution of social complexity in the species to be considered.

## Iconicity

Even if we can suggest factors that might have contributed to the elaboration of complexity in vocal (and gestural) expression and can see possible models for precursors to human speech in phenomena such as vocalized lip-smacking displays in baboons or girneys in Japanese macaques (Green, 1975), the issue of how these gestures acquired symbolic significance still eludes us. It is often maintained that in human speech linguistic symbols are “arbitrary.” This makes it difficult to see how they could have been derived from sounds that have any sort of iconic relationship to the features of their referents. In sign language this is different. Many signs in all sign languages studied can be understood as having developed from actions depictive of object appearance or of actions of some sort (see Taub, 2001). The transitions that we can sometimes observe, in sign language, from pantomimimc or descriptive gestures to arbitrary expressions that conform to the formational constraints of the language system, is seen by many to provide a general model for the process by which linguistic signs come into being.

Though the arbitrary relationship between word form and word meaning has long been the dominant doctrine, there have always been those who have found much evidence for iconicity in spoken language. This claim (as well as its opposite) was discussed in Plato’s *Cratylus*, and the debate has persisted ever since (Genette, 1995). Interest in iconicity in spoken language has begun to grow lately. Taking cues from sign languages, and also from studies of grammaticalization in spoken languages, it seems clear that iconicity is widespread in spoken languages, as it is in sign languages. Some, such as Talmy Givon, consider it fundamental. Thus Givon (1985, p. 214) has written, “for us to understand the ‘magic’ of symbolic representation, we ought to consider iconicity the truly general case in the coding, representation and communication of experience and [arbitrary] symbols as a mere extreme case on the iconic scale.” Modes of expressive action, whether kinesic or oral–aural, all have their iconic potential. This appears to be especially exploited when new expressions are being formed. The features that can be expressed iconically, however, will be different, according to whether the medium is oral–aural or kinesic. Engaging in actions (vocal or kinesic) that depict features of what is being referred to is fundamental to how either sounds or actions can be made as representations. That is, the ability to engage in *mimesis* (and of course, in its reciprocal, the ability to recognize an action or a sound *as mimetic*) is what made the development of language possible (Donald, 1991).

Contrary to what has often been maintained, there is support for the view that the kinesic medium is not markedly favored, in this respect, over the oral–aural medium. Evidence from studies of sound symbolism (Hinton, Nichols, & Ohala, 1994), the mimetic and depictive potentials of the nonverbal capacities of the vocal medium (Perlman, Dale, & Lupyan, 2015), studies of *ideophones* (Voeltz & Kilian-Hatz, 2001), and the example of so-called *mimetics* in Japanese (Hamano, 1998), among much else, supports the notion that iconicity is as fundamental a feature of the vocal as of the kinesic modality (see also Dingemanse et al., 2015; Nuckolls, 1999; Perniss & Vigliocco, 2014). This is less apparent in spoken than in sign languages partly because spoken languages are so very ancient. Once spoken languages begin to develop—building words out of existing words through all the potentials for sound recombinations offered by phonology, acquiring and modifying words from other languages, and so on—and given the pervasiveness of meaning extension, iconicity can be obscured or overlaid. These processes occur in sign languages also, but communities of signers are rare, and hitherto have not lasted for very long periods. Consequently, there has probably been insufficient time for these processes to have had as pervasive effects as they have in spoken languages.

I conclude, thus, that the mimetic process is common and important for both the vocal and kinesic modalities. If this is the case, a “gesture-first” theory of language emergence may be unnecessary.

## Why speakers mobilize their hands when speaking

However, there remains the issue of why, in speakers, the hands and other bodily articulators are often mobilized when someone engages in utterances. Work on speaker gestures, investigating how they relate to speech (see Kendon, 2004; McNeill, 1992, 2000), has shown that gesturing and speaking are components of a single process of utterance generation. Gesturing must be considered as much a part of languaging as speaking is. As the brief review above of the evidence called to support “gesture first” has shown, it does not make a compelling case for “gesture first,” but it does make a compelling case *that gesturing is a part of languaging.* An account of why this is so will be needed if we are to explain language evolution.

A survey of examples, taken from video-recordings made at various kinds of interactional occasions, shows that these speaker manual actions do many different things (see Kendon, 2004, chaps. 9–10). For example, they serve to provide dimensional and dynamic information about the objects or actions that a speaker is talking about. They can give information about the relative dimensions, shape, or spatial positioning of nominated objects, or they may refer to the manner of the action named by a verb. Entities created as “virtual entities” through manual action can be moved about or placed in relation to one another, or the hands can create visual diagrams or movement pattern demonstrations, in this way giving visible form to abstract relationships referred to in speech. Such use shows, in terms of visible actions, the propensity for concepts to be derived from our experience and interactions with the physical environment, its contents, and our spatial conception of it. This supports the view that we use the experiences of our body and how we operate with it in the physical world as a framework for thinking about things that are more abstract (Cienki & Müller, 2008).

It should also be noted that many of the manual movements that speakers make seem to be enactments, albeit highly schematized, of the illocutionary forces of the units of spoken discourse. As speech act theory proposes, any act of speaking is also a mode of action. Thus in saying something one may *assert*, *request*, *deny*, *withdraw*, *hold up*, *stop*, *offer*, *present*, *indicate*, and a host of other actions. Very often the manual actions associated with speaking express these kinds of “pragmatic meanings” rather than information related to the propositional content of the discourse (Kendon, 2004, chaps. 11–13; Streeck, 2009, chap. 8).

Speaker manual actions, thus, are a part of utterances both in terms of action and at the level of conceptual expression; they are an integral part of what is being said and done when someone is languaging. Their forms of action, understood as referring to conceptual categories, may enter directly into the structure of the utterance, or they may also display what *kind* of a speech action or move is being done with an utterance*.*

How are gestures recognized as meaningful in these ways? As was already suggested, they are forms of action that—although schematized, abbreviated, and often conventionalized—tend to be recognized as derived from forms of practical action, such as sketching, object manipulation, brushing away, smoothing a surface, grasping an object, doing something with an object, and the like (Müller, 2014). In my view, this suggests that gestures can best be understood as forms of action derived from how one uses one’s hands to show or change the shape or form of things—to pick things up, let them drop from one’s hands, place one’s hands around an object, grasp an object, do something with an object, carry out patterns of action, and so forth. It seems they are understood exactly *as* actions of this sort, and that, once a person is able to perform the action, he or she can arrive at its aim, providing a clue to the concept (or class of concept) that the gesture is used to conjure up.

David McNeill (2016) opposes the view offered here on the grounds that gestures cannot combine with speech if they have real-world practical aims. However, in my view, gestures, like speech, operate to conjure forth virtual worlds, which are the worlds we inhabit when languaging. Words and gestures labor *together* to produce virtual objects that serve as conceptual expressions. The co-involvement of gesturing with speech—where gestures are schematic forms abstracted from practical action—indicates that languaging is derived from practical action. This leads to the suggestion that speaking, like co-occurring manual gesturing, is manipulatory activity in the abstract. The hands and the mouth, as executive organs, intimately linked as they are, work in conjunction when the individual is engaged in acting on the world and interacting with other beings. Accordingly, these executive organs are likewise mobilized when the world is virtual, as when we language.
